# Metabolic Kinases as Regulators of Inter‐Organelle Communication in Aging and Age‐Related Diseases

**DOI:** 10.1111/acel.70624

**Published:** 2026-07-05

**Authors:** Md Riad Chowdhury, Jae‐Han Jeon, Dipanjan Chanda

**Affiliations:** ^1^ Department of Biomedical Science, Graduate School Kyungpook National University Daegu Republic of Korea; ^2^ Department of Internal Medicine, School of Medicine Kyungpook National University Chilgok Hospital, Kyungpook National University Daegu South Korea; ^3^ Research Institute of Aging and Metabolism Kyungpook National University Daegu South Korea

**Keywords:** age‐related diseases, aging, inter‐organelle communication, metabolic kinases, mitochondrial quality control

## Abstract

Cellular aging is accompanied by progressive alterations in metabolic homeostasis, stress adaptation, and organelle function. Increasing evidence suggests that functional coordination among membrane‐bound organelles, including mitochondria, the endoplasmic reticulum (ER), lysosomes, peroxisomes, and the Golgi apparatus, contributes to cellular homeostasis during aging. However, the mechanisms linking kinase signaling to specific inter‐organelle contact sites or communication pathways remain incompletely defined. In this review, we discuss current evidence linking major metabolic and stress‐responsive kinases, including AMPK, pyruvate dehydrogenase kinases (PDKs), mTOR, AKT, and PERK, to organelle coordination in aging and age‐related diseases. These kinases regulate mitochondrial dynamics, metabolic flux, calcium and lipid handling, autophagy, lysosomal function, proteostasis, and vesicular trafficking. In some contexts, kinase signaling intersects with defined organelle interfaces, such as mitochondria‐associated ER membranes, whereas in many cases the effects on inter‐organelle communication are indirect or inferred from broader changes in organelle function. We further discuss how kinase dysregulation may contribute to age‐associated defects in mitochondria–ER, mitochondria–lysosome, mitochondria–peroxisome, and ER–Golgi coordination in neurodegeneration, cardiometabolic disease, cellular senescence, and inflammaging. By distinguishing direct contact‐site regulation from indirect functional coordination, this review highlights kinase‐regulated organelle communication as an emerging, but still incompletely resolved, framework for understanding cellular decline during aging.

## Graphical abstract

Metabolic kinases integrate cellular energy, nutrient, and stress signals to regulate inter‐organelle coordination during aging. In young or healthy cells, coordinated communication among mitochondria, the endoplasmic reticulum, lysosomes, peroxisomes, and the Golgi apparatus supports calcium handling, lipid metabolism, mitochondrial dynamics, autophagy, redox balance, proteostasis, and vesicular trafficking. These processes are shaped by kinase signaling pathways including AMPK, PDKs, mTOR, AKT, and PERK, primarily through regulation of organelle function rather than direct control of membrane contact‐site formation. During aging and age‐related disease, kinase dysregulation contributes to impaired mitochondria–ER coupling, defective mitochondria–lysosome coordination, disrupted mitochondria–peroxisome function, and compromised ER–Golgi trafficking. This breakdown of organelle coordination promotes mitochondrial dysfunction, oxidative stress, impaired quality control, metabolic imbalance, and cellular decline.

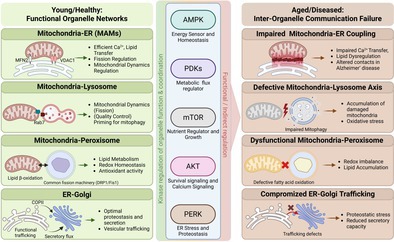

## 1 Introduction

Aging is a multifactorial biological process characterized by the progressive decline of cellular and physiological functions, leading to increased susceptibility to chronic diseases. While traditional frameworks have emphasized damage to individual cellular components, recent studies highlight that cellular homeostasis depends on coordinated interactions between membrane‐bound organelles, including mitochondria, the endoplasmic reticulum (ER), lysosomes, peroxisomes, and the Golgi apparatus (Huang et al. 2022; Li et al. 2024; Silva et al. 2020). These organelles do not operate in isolation but form an interconnected network that enables the exchange of metabolites, lipids, calcium, and stress signals, allowing cells to dynamically adapt to environmental and metabolic changes (Silva et al. 2020).

Inter‐organelle communication occurs through multiple mechanisms, including vesicular trafficking and physical membrane contact sites such as mitochondria‐associated ER membranes (MAMs), mitochondria‐lysosome interfaces, and peroxisome‐ER junctions (Chen et al. 2020; Morciano et al. 2018; Wong et al. 2019). These contact sites function as specialized signaling platforms that regulate metabolic flux, calcium homeostasis, and organelle quality control. Importantly, alterations in organelle communication have been associated with mitochondrial dysfunction, ER stress, impaired autophagy, and metabolic imbalance observed during aging and in age‐related diseases (López‐Otín et al. 2023; Picca et al. 2020). However, it is important to distinguish direct regulation of contact‐site dynamics from broader changes in organelle function, as these processes are mechanistically distinct.

Within this framework, metabolic kinases have emerged as key signaling nodes that integrate nutrient availability, energy status, and stress responses (Hardie et al. 2012; Saxton and Sabatini 2017; Schröder and Kaufman 2005). Kinases such as AMPK, mTOR, and AKT act as central regulators of metabolic homeostasis, whereas PERK and pyruvate dehydrogenase kinases (PDKs) represent stress‐responsive and metabolic flux‐regulating kinases, respectively (Hardie et al. 2012; Ricoult and Manning 2013; Saxton and Sabatini 2017; Schröder and Kaufman 2005; Zhang et al. 2014). These signaling pathways influence organelle behavior through phosphorylation‐dependent mechanisms that regulate mitochondrial dynamics, autophagy, lipid metabolism, and proteostasis (Hardie et al. 2012; Hu et al. 2021; Saxton and Sabatini 2017). In some contexts, kinases have been reported to act directly at organelle interfaces or contact‐site‐associated complexes, whereas in others they influence communication indirectly by altering metabolic or stress states (Verfaillie et al. 2012).

Importantly, the evidence supporting kinase‐dependent regulation of inter‐organelle communication is uneven across organelle systems and disease contexts. In some cases, kinases have been linked to defined contact‐site‐associated proteins or signaling mediators, whereas in many others their effects on organelle communication are inferred from broader changes in organelle function, metabolic state, autophagy, or stress signaling (Betz et al. 2013; Verfaillie et al. 2012; Wong et al. 2018). Therefore, throughout this review, we distinguish established contact‐site regulations from indirect functional coordination and highlight areas where direct causality remains unresolved. This distinction is particularly important in aging biology, where findings from physiological aging models, progeroid conditions, and age‐related disease contexts may reflect overlapping but nonidentical mechanisms.

In this review, we discuss how metabolic and stress‐responsive kinases regulate inter‐organelle communication, with a focus on mitochondria–ER, mitochondria–lysosome, mitochondria–peroxisome, and ER–Golgi networks. We further discuss how aging‐associated dysregulation of kinase signaling contributes to impaired organelle communication and the development of age‐related diseases. By distinguishing direct and indirect mechanisms of regulation, this review aims to provide a more precise framework for understanding how kinase signaling integrates organelle communication in aging.

## 2 Metabolic Kinases as Central Integrators of Cellular Homeostasis

Cellular homeostasis depends on the coordinated regulation of metabolic activity, stress responses, and organelle function (López‐Otín et al. 2023; Silva et al. 2020). Metabolic kinases act as central signaling nodes that integrate nutrient availability, energy status, and proteostatic stress to regulate these processes (Hardie et al. 2012; Saxton and Sabatini 2017). Through phosphorylation‐dependent mechanisms, they influence mitochondrial metabolism, ER stress signaling, lysosomal function, and cellular biosynthetic output, thereby shaping inter‐organelle communication networks (Hardie et al. 2012; Hu et al. 2021; Saxton and Sabatini 2017; Verfaillie et al. 2012).

### 2.1 AMPK

AMP‐activated protein kinase (AMPK) is a central energy sensor that responds primarily to changes in the AMP/ATP ratio, linking cellular energetic stress to downstream organelle remodeling and metabolic adaptation (Hardie et al. 2012). In the context of inter‐organelle communication, AMPK is best supported as a regulator of mitochondrial dynamics, autophagy, lysosomal function, and metabolic adaptation (Hardie et al. 2012). Whether AMPK directly regulates specific membrane tethering complexes remains insufficiently studied, and current evidence more strongly supports an indirect role in shaping organelle coordination through these broader functional pathways. At mitochondria–ER interfaces, AMPK phosphorylates mitochondrial fission factor (MFF), promoting DRP1‐dependent mitochondrial fission and remodeling of mitochondria‐associated ER membranes (MAMs) under energetic stress (Hu et al. 2021).

AMPK also regulates mitochondrial quality control through phosphorylation of ULK1, thereby initiating autophagy (Egan et al. 2011), and through activation of TFEB‐dependent lysosomal biogenesis pathways (Saxton and Sabatini 2017; Settembre et al. 2011). In addition, AMPK has been implicated in the regulation of lysosomal function through pathways involving phosphoinositide metabolism, including PIKfyve‐mediated endolysosomal dynamics and membrane trafficking (Ikonomov et al. 2015). However, it is important to note that these mechanisms primarily influence organelle interaction indirectly by modulating autophagy and lysosomal function, rather than directly regulating the formation or stability of mitochondria‐lysosome contact sites.

In addition, AMPK has been implicated in the regulation of organelle positioning and metabolic adaptation through effects on cytoskeletal dynamics and lipid metabolism (Mihaylova and Shaw 2011). Collectively, these findings position AMPK as a key integrator of metabolic stress signals that shape inter‐organelle communication predominantly through indirect, functionally coupled pathways.

### 2.2 PDKs

Pyruvate dehydrogenase kinases (PDKs) are key regulators of mitochondrial substrate utilization that control the entry of glycolytic carbon into the tricarboxylic acid (TCA) cycle through inhibitory phosphorylation of the pyruvate dehydrogenase complex (Patel et al. 2014; Zhang et al. 2014). By shifting metabolism between glucose oxidation and fatty acid utilization, PDKs play an important role in maintaining metabolic flexibility and regulating mitochondrial redox balance and reactive oxygen species (ROS) production (Zhang et al. 2014).

In contrast to kinases such as AMPK and mTOR, the role of PDKs in inter‐organelle communication is less well defined. Emerging evidence suggests that PDK4 may associate with mitochondria‐associated ER membranes (MAMs) and influence calcium signaling through interaction with components of the IP3R‐GRP75‐VDAC1 complex, thereby modulating ER–mitochondria coupling under stress conditions (Thoudam et al. 2019). However, these findings remain limited, and direct phosphorylation targets of PDKs at organelle contact sites have not been comprehensively established.

More broadly, PDK‐dependent metabolic rewiring can influence organelle communication indirectly by altering mitochondrial function, substrate availability, and redox status. For example, increased PDK activity has been linked to enhanced fatty acid oxidation, mitochondrial stress, and altered ROS signaling, all of which can secondarily affect ER stress responses, autophagy, and lipid metabolism (Zhang et al. 2014). Thus, current evidence supports a model in which PDKs regulate inter‐organelle communication predominantly through modulation of metabolic state rather than direct control of contact‐site architecture.

### 2.3 mTOR

The mechanistic target of rapamycin (mTOR) is a central regulator of cellular growth and metabolism that integrates nutrient availability, energy status, and growth factor signaling (Saxton and Sabatini 2017). mTOR functions through two distinct complexes, mTORC1 and mTORC2, with mTORC1 localized to the lysosomal surface, positioning lysosomes as key signaling hubs within the cell (Kim and Guan 2019; Saxton and Sabatini 2017).

Through phosphorylation of ULK1, mTORC1 suppresses autophagy initiation, while inhibition of TFEB nuclear translocation limits lysosomal biogenesis and degradative capacity (Settembre et al. 2011). These processes regulate lysosomal function and thereby indirectly influence mitochondrial quality control and mitochondria‐lysosome coordination. However, it is important to distinguish these effects on lysosomal capacity from direct regulation of mitochondria‐lysosome contact sites, for which evidence remains limited.

mTORC2 contributes to cytoskeletal organization and spatial distribution of organelles through AKT‐dependent signaling, influencing cell survival and metabolic adaptation (Manning and Toker 2017; Saxton and Sabatini 2017). Collectively, mTOR signaling plays a major role in coordinating organelle function and positioning, but current evidence suggests that its influence on inter‐organelle communication is primarily indirect and mediated through regulation of autophagy, lysosomal activity, and cellular metabolic state rather than direct control of membrane contact site architecture.

### 2.4 AKT

AKT is a central kinase downstream of growth factor signaling that integrates extracellular cues with intracellular metabolic and survival pathways (Manning and Toker 2017). In the context of inter‐organelle communication, AKT has been most clearly implicated in the regulation of signaling at ER–mitochondria interfaces rather than direct control of physical membrane tethering.

At these interfaces, AKT modulates calcium signaling through phosphorylation of key components such as inositol 1,4,5‐trisphosphate receptors (IP3Rs), thereby influencing calcium transfer from the ER to mitochondria and regulating apoptotic sensitivity (Marchi et al. 2012). AKT has also been reported to regulate phosphofurin acidic cluster sorting protein 2 (PACS2), a protein involved in ER–mitochondria coupling, further linking AKT signaling to the functional organization of mitochondria‐associated ER membranes (MAMs) (Simmen et al. 2005).

In addition, AKT influences organelle function more broadly through activation of mTORC1 signaling, thereby regulating protein synthesis, ER homeostasis, and metabolic adaptation (Manning and Toker 2017; Saxton and Sabatini 2017). These roles position AKT as a regulator of inter‐organelle communication primarily through modulation of signaling pathways and calcium flux rather than direct structural control of contact sites.

### 2.5 PERK

Protein kinase RNA‐like ER kinase (PERK) is a key sensor of endoplasmic reticulum (ER) stress and a central component of the unfolded protein response (UPR), linking proteostatic stress to adaptive and apoptotic signaling pathways (Schröder and Kaufman 2005). Unlike classical metabolic kinases, PERK functions primarily as a stress‐responsive kinase localized to the ER membrane.

Importantly, PERK has been shown to localize at mitochondria‐associated ER membranes (MAMs), where it contributes to communication between the ER and mitochondria. At these interfaces, PERK activity is required for the propagation of reactive oxygen species (ROS)–induced apoptotic signaling and for maintaining functional ER–mitochondria coupling under stress conditions (Verfaillie et al. 2012). PERK‐dependent phosphorylation of eIF2α regulates global protein synthesis, thereby linking ER stress to mitochondrial function and cellular adaptation (Harding et al. 2000).

In addition to its role in translational control, PERK has been implicated in the regulation of calcium signaling and redox homeostasis at ER–mitochondria interfaces, further supporting its role in coordinating stress responses between organelles (Verfaillie et al. 2012). These features position PERK as a key mediator of inter‐organelle communication under conditions of proteostatic and oxidative stress, providing a mechanistic link between ER stress signaling and mitochondrial dysfunction.

## 3 Organelle Communication Networks in Aging

### 3.1 Mitochondria‐ER

**FIGURE 1:**
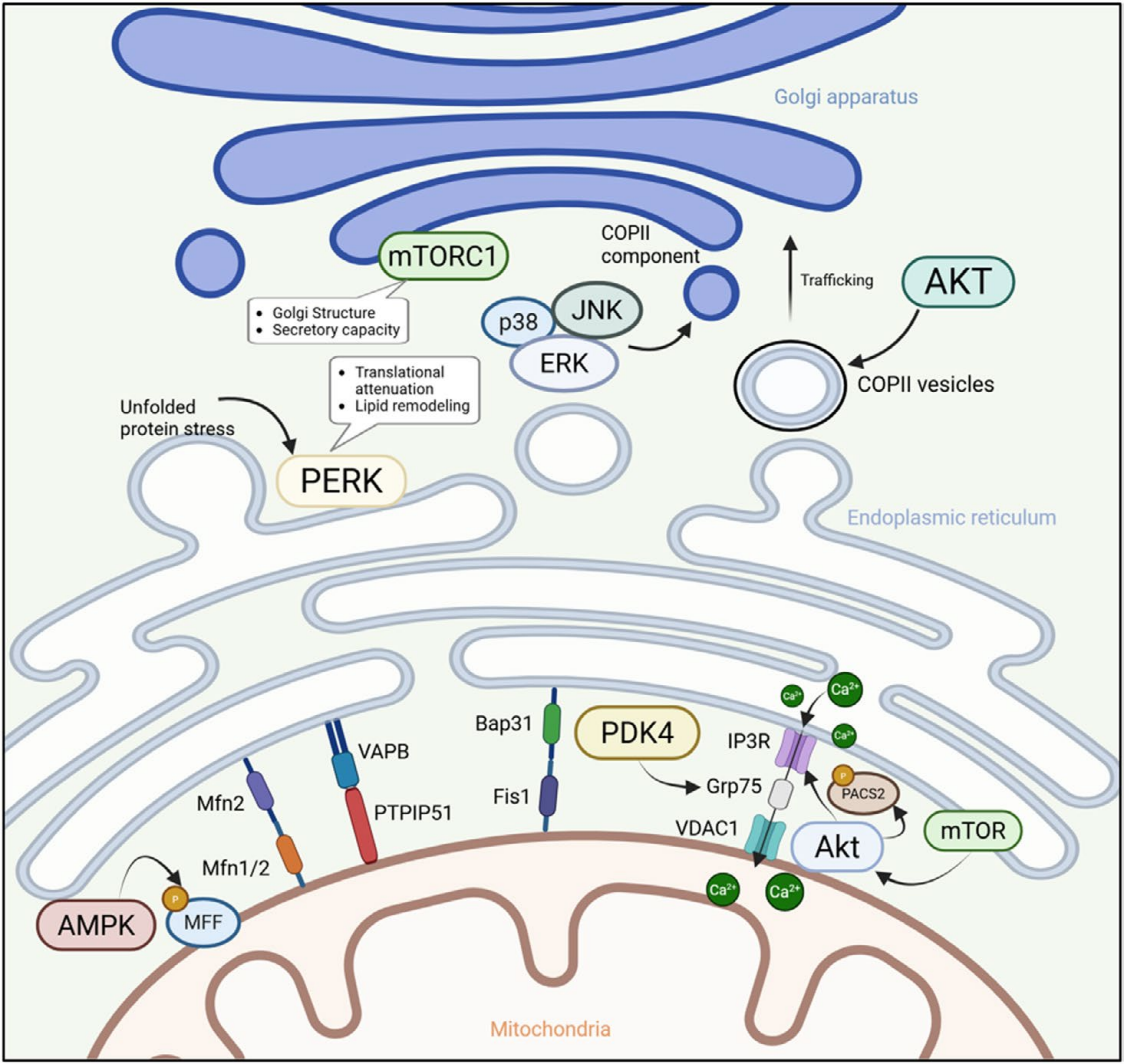
Metabolic kinase regulation of mitochondria‐ER (MAM) and ER–Golgi communication. Mitochondria‐associated ER membranes (MAMs) are formed by tethering complexes including MFN1/2, VAPB–PTPIP51, Fis1–Bap31, and the IP3R‐GRP75‐VDAC1 axis, enabling calcium transfer, lipid exchange, and apoptotic signaling. Metabolic kinases modulate these functions: AMPK promotes mitochondrial fission and MAM remodeling via MFF phosphorylation, whereas AKT regulates calcium signaling and MAM stability through phosphorylation of IP3Rs and PACS2. PDK4 has been reported to associate with MAM components and may influence ER‐mitochondria coupling under metabolic stress. In parallel, ER‐Golgi communication is regulated through vesicular trafficking, where mTORC1 and PERK control protein synthesis and stress responses, and AKT influences trafficking efficiency. These pathways coordinate organelle function primarily through signaling and metabolic regulation rather than direct control of membrane contact site formation.

The structural and functional coupling between the mitochondria and the ER occurs at mitochondria‐associated ER membranes (MAMs) (Y. Liu et al. 2024). Several tethering complexes facilitate the formation and stability of these MAMs, including Mitofusin‐2 (Mfn2)‐Mitofusin‐1/2 (Mfn1/2), vesicle‐associated membrane protein‐associated protein B (VAPB)‐protein tyrosine phosphatase‐interacting protein‐51 (PTPIP51), inositol‐1,4,5‐trisphosphate receptor (IP_3_R3)‐glucose‐regulated protein‐75 (Grp75)‐voltage‐dependent anion channel 1 (VDAC1), and Fission 1 (Fis1)‐B cell‐associated protein 31 (Bap31) (Degechisa et al. 2022) (Figure 1). Through these tethering interactions, MAMs serve as specialized microdomains that regulate various essential cellular functions, including Ca^2+^ transfer, lipid synthesis and signaling, and mitochondrial dynamics (Liu et al. 2024). Beyond these roles, MAMs also serve as critical platforms for phospholipid trafficking, steroidogenesis, and apoptotic signaling (Latino et al. 2024; Morciano et al. 2018).

These communications between mitochondria and ER are regulated by different metabolic kinases. For instance, AMPK, the master energy sensor, initiates metabolic and structural adjustments at the MAMs during energy stress, promoting mitochondrial fission and mitophagy through the phosphorylation of mitochondrial fission factor (MFF) (Hu et al. 2021). On the other hand, AKT helps stabilize MAMs by phosphorylating PACS2 and regulates calcium release through mTORC2‐dependent phosphorylation of IP3R, thereby modulating apoptosis (Betz et al. 2013). Similarly, PDK4 has been reported to be associated with MAM components and may influence ER‐mitochondria coupling through interaction with the IP3R1‐GRP75‐VDAC1 axis, although direct regulation of MAM structure remains to be fully established (Thoudam et al. 2019).

During aging and age‐related disease, ER‐mitochondria communication appears to undergo context‐dependent remodeling rather than a uniform decline. Some studies report reduced ER‐mitochondria coupling associated with impaired calcium transfer, lipid exchange, mitochondrial bioenergetics, and quality control. For example, age‐associated reduction of ER‐mitochondrial contacts in cardiomyocytes has been linked to impaired mitochondrial lipid metabolism and autophagosome formation (Hong et al. 2025; Figure 4). However, other disease contexts, including Alzheimer's disease, show increased or aberrantly stabilized MAM interactions, indicating that altered ER‐mitochondria communication cannot be interpreted as a simple loss‐of‐contact model (Area‐Gomez et al. 2012). Similarly, reduced PERK function in neurodegenerative contexts may weaken ER‐mitochondria stress signaling and impair ROS‐induced apoptotic responses (Morgado‐Cáceres et al. 2022). Collectively, these findings suggest that MAM remodeling in aging and age‐related disease is dynamic, context‐specific, and likely influenced by both kinase signaling and disease‐specific cellular stress states.

### 3.2 Mitochondria‐Lysosome

**FIGURE 2:**
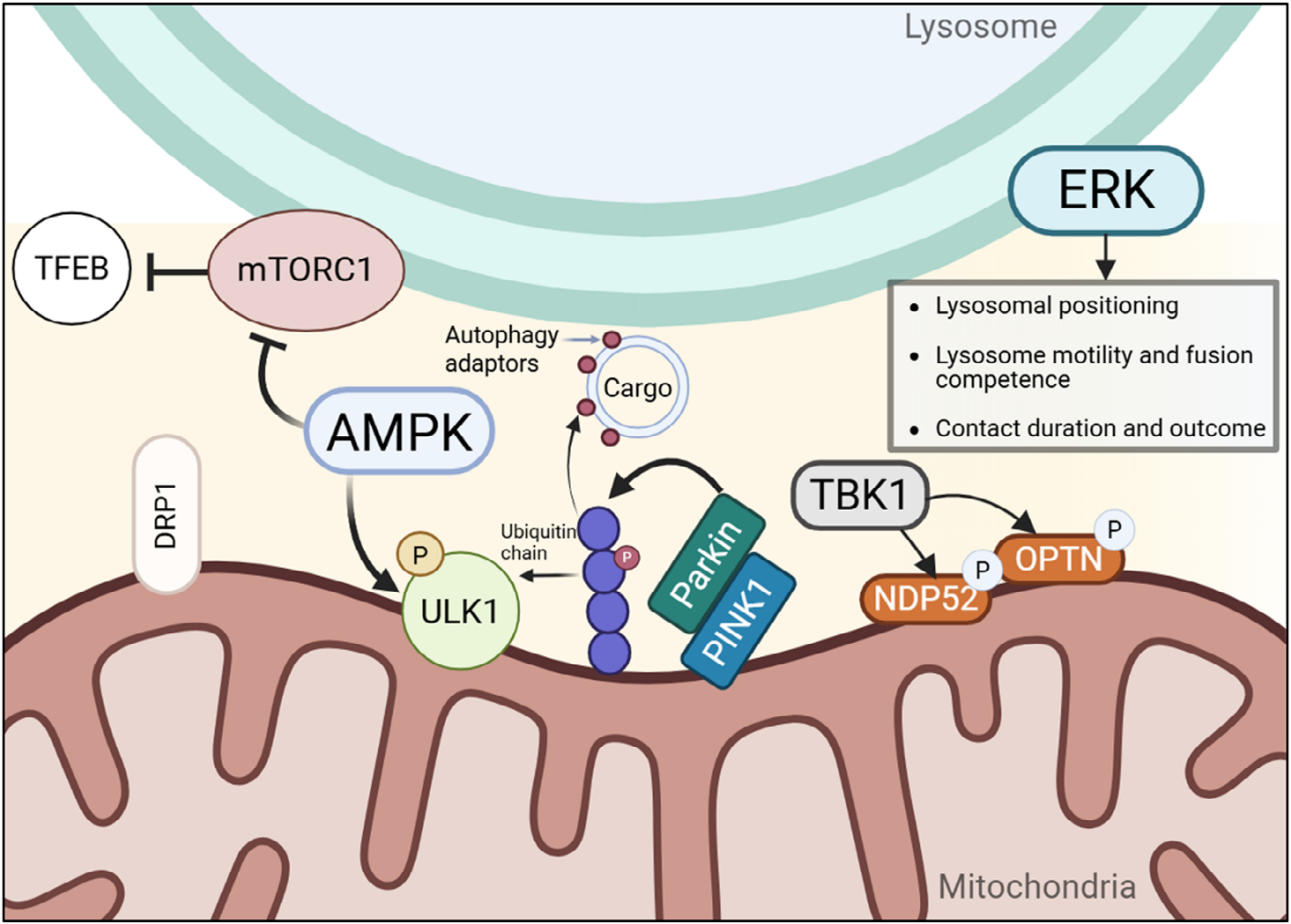
Kinase‐mediated regulation of mitochondria‐lysosome coordination. Mitochondria and lysosomes form transient, non‐degradative contact sites that regulate mitochondrial dynamics independently of autophagy. Rab7‐dependent interactions facilitate DRP1‐mediated mitochondrial fission, enabling segregation of damaged mitochondrial regions. Metabolic kinases regulate this system primarily through control of autophagy and lysosomal function: AMPK promotes autophagy initiation via ULK1 phosphorylation and enhances lysosomal biogenesis through TFEB activation, whereas mTORC1 suppresses these processes under nutrient‐rich conditions. TBK1 enhances mitophagy by phosphorylating autophagy receptors such as OPTN and NDP52, and MAPK/ERK signaling influences lysosomal positioning and fusion dynamics. These pathways modulate mitochondrial turnover and lysosomal capacity but are not established as direct regulators of mitochondria–lysosome contact‐site formation.

Mitochondria‐lysosome communication has emerged as an important component of cellular homeostasis, extending beyond the classical role of lysosomes as terminal degradative compartments. In addition to their role in autophagy, lysosomes form transient, non‐degradative contact sites with mitochondria that regulate mitochondrial dynamics and lysosomal positioning (Wong et al. 2019). These interactions are increasingly recognized as signaling interfaces rather than simply intermediates in the degradative pathway (Figure 2).

Mitochondria‐lysosome contact sites have been shown to coordinate mitochondrial fission events independently of autophagy. Specifically, Rab7‐dependent contacts between mitochondria and lysosomes facilitate DRP1‐mediated mitochondrial fission, enabling the segregation of damaged mitochondrial regions (Wong et al. 2019, 2018). These contacts are dynamic and tightly regulated, with GTP hydrolysis‐dependent Rab7 cycling controlling the formation and resolution of contact events (Wong et al. 2019). Importantly, these processes occur independently of lysosomal degradation and represent a distinct layer of mitochondrial quality control.

While mitophagy ultimately depends on lysosomal degradation, it is mechanistically distinct from mitochondria‐lysosome contact site function. Upon mitochondrial damage, the PINK1‐Parkin pathway promotes ubiquitination of mitochondrial proteins and recruitment of autophagy adaptors, leading to autophagosome formation and subsequent lysosomal fusion (Pickrell and Youle 2015). However, this degradative pathway is temporally and mechanistically separate from the earlier contact‐mediated regulation of mitochondrial dynamics.

Metabolic kinases influence mitochondria‐lysosome coordination primarily through regulation of autophagy and lysosomal function rather than direct control of contact‐site formation. AMPK promotes autophagy initiation through ULK1 phosphorylation and supports lysosomal biogenesis via TFEB signaling, whereas mTORC1 suppresses these processes under nutrient‐rich conditions (Egan et al. 2011; Saxton and Sabatini 2017; Settembre et al. 2011). These pathways modulate mitochondrial turnover and lysosomal capacity but have not been shown to directly regulate the formation of mitochondria‐lysosome contact sites (Figure 2).

During aging, lysosomal function declines, characterized by reduced acidity and impaired degradative capacity, leading to inefficient autophagic flux and accumulation of damaged mitochondria (López‐Otín et al. 2023; Pickrell and Youle 2015). In parallel, age‐associated defects in mitophagy, including reduced responsiveness of the PINK1–Parkin pathway, further contribute to the persistence of dysfunctional mitochondria and increased oxidative stress. Although direct evidence for aging‐associated alterations in mitochondria–lysosome contact sites remains limited, age‐related defects in mitochondrial quality control, lysosomal acidity, and degradative capacity may weaken functional coordination between these organelles. Whether these changes reflect altered contact‐site dynamics, impaired downstream degradation, or broader lysosomal dysfunction remains an important open question.

### 3.3 Mitochondria‐Peroxisome

Mitochondria and peroxisomes cooperate closely in lipid metabolism and redox homeostasis, forming a functionally integrated network that supports cellular metabolic flexibility (Fransen et al. 2017). Peroxisomes initiate the β‐oxidation of very‐long‐chain and branched chain fatty acids, generating shortened lipid intermediates that are subsequently transferred to mitochondria for further oxidation (Schrader et al. 2015; Wanders and Waterham 2006). This functional division enables efficient processing of lipid substrates that cannot be fully metabolized within a single organelle.

In addition to metabolic coordination, mitochondria and peroxisomes contribute to cellular redox balance through shared regulation of reactive oxygen species (ROS) (Fransen et al. 2017). Peroxisomes generate hydrogen peroxide as a byproduct of fatty acid oxidation, while mitochondria are major sources of superoxide and other ROS species. The coordinated activity of antioxidant systems across both organelles is therefore essential to maintain redox homeostasis (Fransen et al. 2017).

Physical and functional interactions between mitochondria and peroxisomes have been described, including shared fission machinery such as DRP1 and Fis1, suggesting coordinated organelle dynamics (Schrader et al. 2015). However, compared with mitochondria–ER or mitochondria–lysosome interfaces, direct evidence for stable or specialized membrane contact sites between mitochondria and peroxisomes remains limited.

**FIGURE 3:**
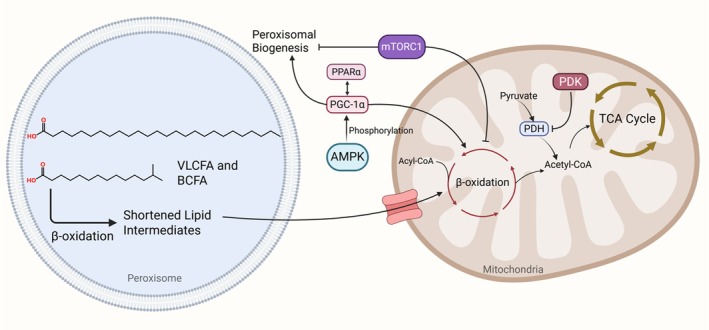
Metabolic regulation of mitochondria–peroxisome coordination. Mitochondria and peroxisomes cooperate in fatty acid metabolism and redox homeostasis. Peroxisomes initiate β‐oxidation of very‐long‐chain fatty acids, generating intermediates that are further metabolized by mitochondria. These organelles also coordinate reactive oxygen species (ROS) metabolism through complementary antioxidant systems. While shared fission machinery such as DRP1 and Fis1 supports coordinated organelle dynamics, direct membrane contact sites between mitochondria and peroxisomes remain less well defined. Metabolic kinases regulate this coordination primarily through control of metabolic pathways: AMPK promotes fatty acid oxidation and peroxisomal biogenesis via PGC‐1α and PPAR signaling, whereas mTORC1 is shown as a broader nutrient‐sensing regulator of metabolic state rather than a direct regulator of mitochondria–peroxisome contact formation. PDKs regulate mitochondrial substrate utilization and metabolic flux, indirectly influencing peroxisomal function.

Metabolic kinases influence mitochondria‐peroxisome coordination primarily through regulation of metabolic pathways rather than direct control of inter‐organelle contact sites. For example, AMPK promotes fatty acid oxidation and peroxisomal biogenesis through PGC‐1α and PPAR signaling pathways. In contrast, mTORC1 is discussed here primarily as a broader nutrient‐sensing regulator of metabolic state rather than as a direct regulator of peroxisome biogenesis (Hardie et al. 2012; Saxton and Sabatini 2017; Schrader et al. 2015). Similarly, PDK‐dependent regulation of mitochondrial substrate utilization alters metabolic flux and redox balance, which may indirectly influence peroxisomal function (Zhang et al. 2014) (Figure 3).

During aging, both mitochondrial and peroxisomal functions are progressively impaired, leading to reduced fatty acid oxidation capacity and accumulation of lipid‐derived and oxidative stress (Fransen et al. 2017; Schrader et al. 2015). Although direct evidence for aging‐associated disruption of mitochondria–peroxisome contact sites remains limited, age‐related declines in metabolic flexibility and antioxidant capacity likely weaken the functional coordination between these organelles. These changes may contribute to lipid dysregulation and redox imbalance observed in aging and metabolic disease.

### 3.4 ER‐Golgi

Communication between the endoplasmic reticulum (ER) and the Golgi apparatus is primarily mediated through vesicular trafficking rather than stable membrane contact sites. Proteins and lipids synthesized in the ER are transported to the Golgi via COPII‐coated vesicles, where they undergo further processing, modification, and sorting before delivery to their final cellular destinations (Barlowe and Miller 2013). This dynamic trafficking system is essential for maintaining protein homeostasis, lipid distribution, and overall secretory function.

In addition to vesicular transport, bidirectional communication between the ER and Golgi supports quality control and stress adaptation. Disruptions in ER proteostasis can impair Golgi function, while defects in Golgi trafficking can lead to accumulation of misfolded proteins within the ER, highlighting the interdependence of these organelles (Appenzeller‐Herzog and Hauri 2006).

Metabolic kinases influence ER‐Golgi communication primarily through regulation of protein synthesis, stress responses, and trafficking pathways rather than direct control of physical contact sites. mTORC1 regulates protein translation and secretory load, whereas PERK modulates translational attenuation during ER stress through phosphorylation of eIF2α (Saxton and Sabatini 2017; Schröder and Kaufman 2005). In addition, AKT signaling has been linked to regulation of COPII vesicle formation and trafficking efficiency, thereby influencing ER‐to‐Golgi transport (Manning and Toker 2017).

During aging, cellular proteostasis and secretory capacity are progressively impaired, which can affect ER‐Golgi trafficking efficiency. Age‐associated declines in protein quality control and increased burden of misfolded proteins have been reported to disrupt ER function and may indirectly impact Golgi processing (López‐Otín et al. 2023). However, direct evidence for aging‐specific disruption of ER‐Golgi communication or vesicular trafficking remains limited. Thus, current evidence suggests that age‐related changes in ER‐Golgi function likely reflect broader defects in proteostasis and cellular stress responses rather than targeted impairment of this communication pathway.

## 4 Metabolic Kinase Dysregulation in Age‐Related Diseases

The disease contexts discussed below should be interpreted as examples of age‐associated pathological remodeling rather than direct equivalents of physiological aging. Where possible, we distinguish evidence derived from aging models from findings obtained in specific disease models because disease‐associated organelle dysfunction may not always reflect mechanisms of normal aging.

### 4.1 Neurodegenerative Diseases

Neurodegenerative diseases are age‐associated disorders characterized by progressive neuronal loss, metabolic dysfunction, and impaired proteostasis. Neurons are highly dependent on efficient metabolic signaling and inter‐organelle communication because of their elevated energetic demands and limited regenerative capacity (Wang and Yang 2024). Increasing evidence suggests that dysregulation of metabolic kinases contributes to neurodegeneration by impairing mitochondrial quality control, lysosomal function, and ER–mitochondria stress signaling (Paillusson et al. 2016; Wong et al. 2018). Whether these alterations reflect direct disruption of inter‐organelle communication pathways or broader organelle dysfunction remains context‐dependent and incompletely resolved.

Dysregulation of mTOR signaling is frequently observed in neurodegenerative disorders and contributes to disease pathology. Chronic mTORC1 activation suppresses autophagy and lysosomal biogenesis, thereby impairing the clearance of damaged mitochondria and protein aggregates (Lee et al. 2023; Palmer et al. 2025). These effects impair mitochondrial turnover and lysosomal degradative capacity, leading to accumulation of dysfunctional, ROS‐generating mitochondria and increased neuronal vulnerability (Fang et al. 2019; Nixon 2013). In parallel, impaired mTORC2 signaling affects cytoskeletal organization and mitochondrial trafficking, further compromising neuronal homeostasis (Schwarz 2013).

AMPK signaling is also dysregulated in neurodegeneration in a context‐dependent manner. While transient AMPK activation supports mitochondrial biogenesis and autophagy, hyperactivation can impair synaptic function and energy balance (Belforte et al. 2021; Hu et al. 2021). These effects primarily reflect altered metabolic and autophagic regulation rather than direct control of inter‐organelle contact sites.

In Parkinson's disease, defective PINK1‐Parkin‐mediated mitophagy illustrates impaired coupling between mitochondrial damage recognition and lysosomal degradation (Pickrell and Youle 2015). However, whether this reflects altered mitochondria‐lysosome contact‐site dynamics, broader lysosomal dysfunction, or impaired downstream degradative capacity remains incompletely resolved. This defect reflects impaired coupling between mitochondrial damage recognition and lysosomal degradation rather than disruption of physical contact sites per se. Kinase imbalance further compromises this pathway, reinforcing mitochondrial dysfunction and oxidative stress. Additionally, sustained activation of ER stress‐associated kinases, such as PERK, links proteostatic stress to altered ER‐mitochondria calcium signaling and redox imbalance, promoting mitochondrial depolarization and apoptotic susceptibility (Paillusson et al. 2016).

Collectively, current evidence indicates that kinase dysregulation in neurodegenerative diseases affects mitochondrial quality control, lysosomal function, ER stress signaling, and metabolic adaptation. Whether these alterations directly disrupt inter‐organelle communication or reflect broader organelle dysfunction remains an important unresolved question. This gap supports the need for future studies that directly measure organelle contact dynamics and functional signaling between mitochondria, lysosomes, and the ER in disease‐relevant models.

### 4.2 Cardiovascular and Metabolic Aging

Cardiovascular and metabolic diseases are prominent age‐related disorders characterized by impaired mitochondrial function, reduced metabolic flexibility, and abnormal stress adaptation (Lesnefsky et al. 2001; Li et al. 2026). Tissues such as the heart, liver, and skeletal muscle rely heavily on coordinated metabolic kinase signaling to maintain energetic homeostasis, mitochondrial function, and organelle coordination. With aging, dysregulation of these kinases contributes to functional uncoupling between organelles rather than discrete loss of contact sites, ultimately promoting tissue dysfunction.

A central feature of cardiovascular and metabolic aging is disruption of the AMPK‐PDK‐mTOR axis, which governs mitochondrial substrate utilization and quality control (Hardie et al. 2012; Saxton and Sabatini 2017; Zhang et al. 2014). Reduced AMPK activity impairs mitochondrial biogenesis and autophagy, while increased PDK expression, particularly PDK4, shifts metabolism toward fatty acid oxidation, reducing energetic efficiency and increasing oxidative stress (Hardie et al. 2012; Zhang et al. 2014). Concurrently, sustained mTORC1 activation inhibits autophagy and lysosomal biogenesis, impairing mitochondrial turnover and promoting accumulation of dysfunctional mitochondria (Saxton and Sabatini 2017).

In addition, defective mitochondria‐ER communication exacerbates metabolic stress, particularly in cardiomyocytes (Gao et al. 2020). Altered kinase signaling at ER‐mitochondria interfaces perturbs calcium and lipid exchange, linking metabolic imbalance to ER stress and mitochondrial dysfunction, thereby increasing susceptibility to apoptosis (Gao et al. 2020). In insulin resistance and type 2 diabetes, altered AKT–mTOR signaling impairs adaptive autophagy, ER homeostasis, and lipid handling, which may secondarily affect coordination among mitochondria, the ER, and lysosomes (Rieusset 2018; Saxton and Sabatini 2017). These findings are consistent with emerging evidence linking systemic metabolic imbalance, including intestinal energy crisis and altered lipoprotein signaling, to chronic inflammation and metabolic aging (Li et al. 2026).

Collectively, cardiometabolic diseases reflect context‐specific amplification of kinase‐driven metabolic imbalance, which may secondarily affect organelle coordination. These findings highlight metabolic kinases as central mediators linking aging‐associated signaling imbalance to mitochondrial dysfunction, metabolic inflexibility, and tissue degeneration.

### 4.3 Cellular Senescence and Inflammaging

Cellular senescence is an age‐associated stress response characterized by stable cell cycle arrest, metabolic remodeling, and chronic inflammatory signaling (Li et al. 2026; López‐Otín et al. 2013). Senescent cells exhibit pronounced dysfunction of mitochondria, lysosomes, and the ER, indicating that altered organelle coordination may contribute to senescence and inflammaging (López‐Otín et al. 2013; Marchi et al. 2023). In this context, metabolic kinase dysregulation reinforces persistent stress signaling rather than initiating organelle dysfunction.

Chronic mTORC1 signaling in senescent cells inhibits autophagy and lysosomal biogenesis, leading to defective mitochondrial turnover and reduced lysosomal degradative capacity (Carroll et al. 2017). The resulting accumulation of dysfunctional, ROS‐producing mitochondria amplifies oxidative stress and reinforces the senescence‐associated secretory phenotype (SASP) (Correia‐Melo et al. 2016). This is further supported by emerging evidence linking systemic metabolic stress and lipid imbalance to chronic inflammatory signaling in aging (Li et al. 2026). At the same time, dysregulated AMPK signaling fails to adequately restore energy balance or autophagic capacity, further exacerbating metabolic stress (Hardie et al. 2012).

Additionally, sustained activation of ER stress–associated kinases such as PERK disrupts ER–mitochondria calcium signaling and redox homeostasis, stabilizing pro‐inflammatory and apoptosis‐resistant senescent states (Salminen and Kaarniranta 2010; Verfaillie et al. 2012). These changes promote persistent inter‐organelle signaling imbalance that sustains the senescent phenotype.

**FIGURE 4:**
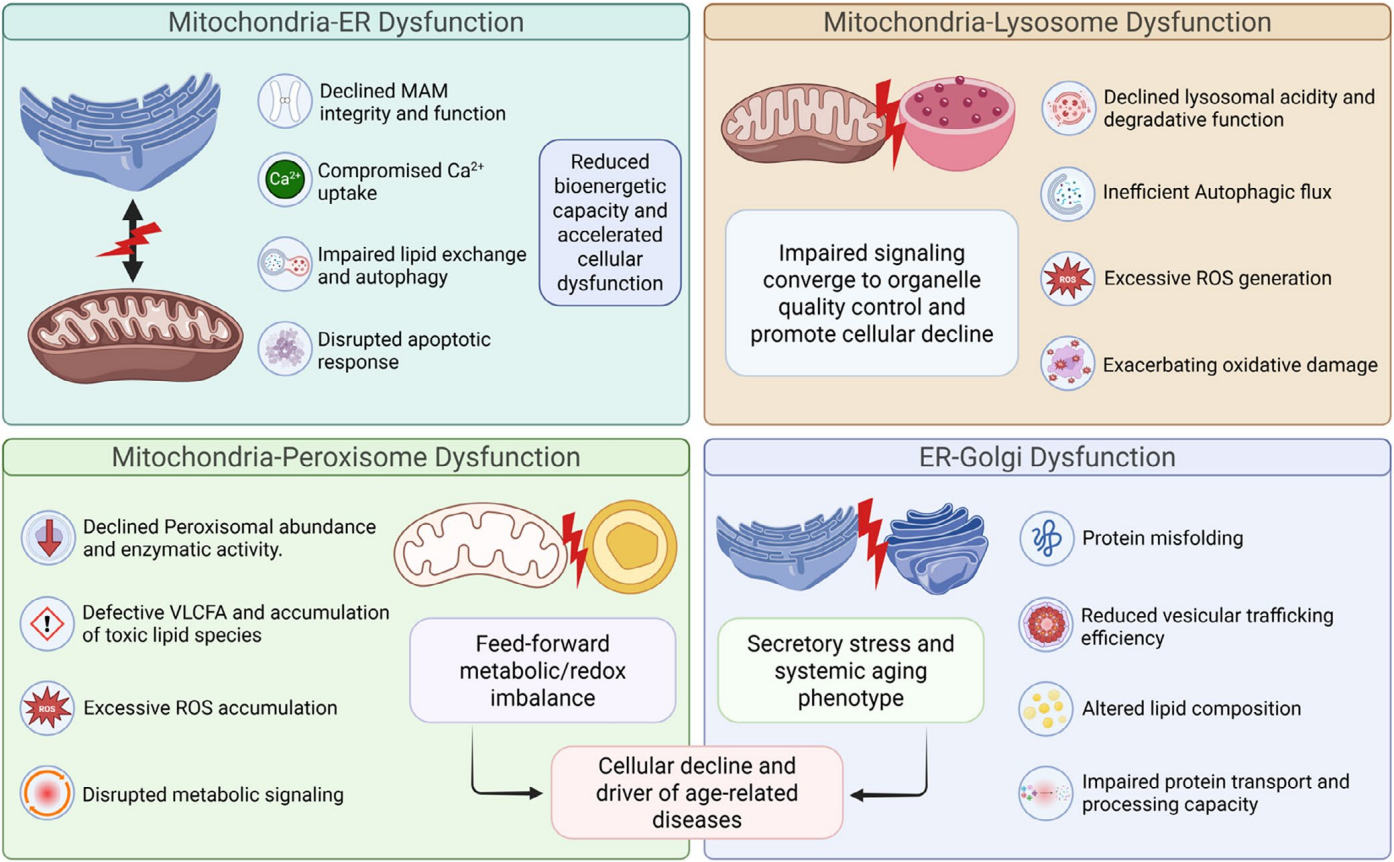
Disruption of inter‐organelle communication during aging. Aging is associated with progressive impairment of organelle function and coordination across inter‐organelle networks. Reduced mitochondria–ER coupling impairs calcium transfer, lipid exchange, and mitochondrial bioenergetics. Declining lysosomal function leads to impaired autophagic flux and accumulation of dysfunctional mitochondria. Disrupted mitochondria–peroxisome coordination contributes to defective fatty acid metabolism and redox imbalance. In parallel, impaired ER–Golgi trafficking reflects broader proteostatic stress and reduced secretory capacity. Together, these changes reflect a systems‐level breakdown of coordinated organelle function, driven by metabolic imbalance, chronic stress signaling, and impaired quality control mechanisms.

Together, these findings suggest that cellular senescence and inflammaging involve chronic kinase‐mediated metabolic and stress signaling imbalance, which may reinforce altered coordination among mitochondria, lysosomes, and the ER. However, direct causal links between specific contact‐site defects and senescent phenotypes remain to be established (Figure 4).

## 5 Emerging Concepts and Future Directions

Despite growing recognition of inter‐organelle communication as a key regulator of cellular homeostasis, several conceptual and technical challenges remain. A major limitation in the field is the difficulty in distinguishing direct regulation of organelle contact sites from indirect effects mediated by global changes in organelle function or metabolic state. Many kinase pathways influence autophagy, mitochondrial dynamics, or lipid metabolism, making it challenging to determine whether observed changes in organelle communication reflect specific alterations in contact‐site architecture or secondary consequences of broader cellular remodeling (Csordás et al. 2018).

A particularly important unresolved question is whether altered inter‐organelle communication acts as a causal driver of aging and disease, an adaptive response to cellular stress, or a downstream consequence of organelle dysfunction. Addressing this issue will require experimental designs that directly measure contact‐site dynamics, inter‐organelle signaling flux, and organelle function in parallel. Such approaches will be essential to determine whether kinase‐dependent changes in organelle coordination are mechanistically upstream of cellular aging phenotypes or instead reflect secondary remodeling.

Another critical challenge lies in the limited availability of tools to selectively manipulate inter‐organelle contact sites. Current approaches, including proximity labeling, split‐fluorescence reporters, and synthetic tethering systems, allow visualization and perturbation of organelle contacts but often lack specificity or may disrupt normal organelle function (Rhee et al. 2013; Ummethum and Hamperl 2020). The development of strategies that can selectively modulate contact‐site components without altering global organelle physiology will be essential to establish causal relationships between kinase signaling and inter‐organelle communication.

The field also faces important context‐dependent complexities. For example, AMPK activation has been reported to exert both protective and detrimental effects in neurodegenerative diseases, depending on context, duration, and disease stage (Vingtdeux et al. 2011; Wang et al. 2019). Similarly, mTOR inhibition is widely considered beneficial for promoting autophagy and longevity, yet chronic suppression of mTOR signaling can impair cellular growth, immune function, and metabolic adaptation (Johnson et al. 2013; Saxton and Sabatini 2017). These context‐dependent effects highlight the need for a more nuanced understanding of how kinase signaling influences organelle coordination across cell types, disease stages, and metabolic states.

An additional layer of complexity arises from cell‐type and tissue‐specific differences in organelle communication networks. Neurons, cardiomyocytes, hepatocytes, and immune cells exhibit distinct metabolic demands and organelle architectures, which shape how kinase signaling pathways influence inter‐organelle interactions (Spinelli and Haigis 2018). For example, neurons rely heavily on mitochondrial trafficking and localized energy production, whereas cardiomyocytes depend on tightly coordinated calcium exchange between the ER and mitochondria. Understanding these cell‐specific regulatory mechanisms will be critical for translating fundamental insights into disease‐relevant contexts.

From a translational perspective, targeting metabolic kinases represents a promising strategy to restore organelle communication in aging and disease. Pharmacological modulators of AMPK, mTOR, and related pathways are already in clinical use or under investigation (Cantó and Auwerx 2010). However, therapeutic targeting of these kinases is complicated by their pleiotropic effects and the risk of systemic metabolic disruption. A key future direction will be the development of approaches that selectively modulate kinase signaling at specific organelle interfaces or within defined cellular contexts.

Emerging technologies, including high‐resolution imaging, spatial proteomics, and single‐cell multi‐omics, are expected to further advance the field by enabling more precise mapping of organelle contact sites and their regulatory networks (Chen et al. 2015; Marx 2021). Integration of these approaches with functional studies will help to distinguish direct signaling mechanisms from secondary effects and refine our understanding of how metabolic kinases coordinate inter‐organelle communication.

Collectively, these challenges and opportunities highlight the need for a more precise and context‐dependent framework for studying inter‐organelle communication. Future studies integrating mechanistic, spatial, and systems‐level approaches will be essential to fully elucidate how kinase signaling networks regulate organelle connectivity and how these processes can be targeted to mitigate aging and age‐related diseases.

## 6 Conclusions

Aging is increasingly recognized as a systems‐level failure of cellular coordination rather than the accumulation of isolated organelle defects. This review highlights metabolic kinases as central integrators of inter‐organelle communication, linking nutrient sensing and stress signaling to the dynamic regulation of mitochondria, the ER, lysosomes, peroxisomes, Golgi, and related contact sites. Disruption of these kinase‐regulated networks emerges as a unifying mechanism underlying mitochondrial dysfunction, impaired quality control, chronic inflammation, and cellular senescence across age‐related diseases.

Considering metabolic kinases as regulators of organelle communication, rather than only as metabolic enzymes, provides a more integrated understanding of aging. This perspective highlights disrupted communication between organelles as a key contributor to cellular decline. Understanding how these signaling networks change with age and differ across tissues will be important for explaining why some cells are more vulnerable to aging. Ultimately, restoring proper communication between organelles may help maintain cellular homeostasis and slow functional deterioration during aging.

## Author Contributions

The original draft was written by M.R.C., and conceptualization was done by M.R.C. and D.C. Review and editing of the manuscript was done by D.C. and J.‐H.J. Visuals are designed by M.R.C., D.C. and J.‐H.J.

## Funding

This work was supported by National Research Foundation of Korea (NRF), RS‐2024‐00352513, RS‐2024‐00450829. Korea Health Technology R&D, RS‐2024‐00437643, RS‐2025‐25460277, RS‐2022‐KH130590.

## Conflicts of Interest

The authors declare no conflicts of interest.

## Data Availability

This is a review paper with no original data. Therefore, data repository submission is not applicable in this case.
